# Machine Learning to Predict Long-Term Cardiac-Relative Prognosis in Patients With Extra-Cardiac Vascular Disease

**DOI:** 10.3389/fcvm.2021.771504

**Published:** 2021-11-25

**Authors:** Guisen Lin, Qile Liu, Yuchen Chen, Xiaodan Zong, Yue Xi, Tingyu Li, Yuelong Yang, An Zeng, Minglei Chen, Chen Liu, Yanting Liang, Xiaowei Xu, Meiping Huang

**Affiliations:** ^1^Department of Radiology, Guangdong Provincial People's Hospital, Guangdong Academy of Medical Sciences, Guangzhou, China; ^2^Department of Radiology, Shenzhen Children's Hospital, Shenzhen, China; ^3^School of Computers, Guangdong University of Technology, Guangzhou, China; ^4^Department of Radiology, The Third Affiliated Hospital, Sun Yat-sen University (SYSU), Guangzhou, China; ^5^School of Medicine, South China University of Technology, Guangzhou, China; ^6^Guangdong Provincial Key Laboratory of South China Structural Heart Disease, Guangdong Cardiovascular Institute, Guangdong Provincial People's Hospital, Guangdong Academy of Medical Sciences, Guangzhou, China; ^7^The Second School of Clinical Medicine, Southern Medical University, Guangzhou, China; ^8^Shantou University Medical College, Shantou, China; ^9^Southern Medical University, Guangzhou, China; ^10^Department of Catheterization Lab, Guangdong Cardiovascular Institute, Guangdong Provincial Key Laboratory of South China Structural Heart Disease, Guangdong Provincial People's Hospital, Guangdong Academy of Medical Sciences, Guangzhou, China

**Keywords:** coronary artery disease, coronary computed tomography angiography, machine learning, prognosis, extravascular disease

## Abstract

**Aim:** Patients with ischemic stroke (IS), transient ischemic attack (TIA), and/or peripheral artery disease (PAD) represent a population with an increased risk of coronary artery disease. Prognostic risk assessment to identify those with the highest risk that may benefit from more intensified treatment remains challenging. To explore the feasibility and capability of machine learning (ML) to predict long-term adverse cardiac-related prognosis in patients with IS, TIA, and/or PAD.

**Methods:** We analyzed 636 consecutive patients with a history of IS, TIA, and/or PAD. All patients underwent a coronary CT angiography (CCTA) scan. Thirty-five clinical data and 34 CCTA metrics underwent automated feature selection for ML model boosting. The clinical outcome included all-cause mortality (ACM) and major adverse cardiac events (MACE) (ACM, unstable angina requiring hospitalization, non-fatal myocardial infarction (MI), and revascularization 90 days after the index CCTA).

**Results:** During the follow-up of 3.9 ± 1.6 years, 21 patients had unstable angina requiring hospitalization, eight had a MI, 23 had revascularization and 13 deaths. ML demonstrated a significant higher area-under-curve compared with the modified Duke index (MDI), segment stenosis score (SSS), segment involvement score (SIS), and Framingham risk score (FRS) for the prediction of ACM (ML:0.92 vs. MDI:0.66, SSS:0.68, SIS:0.67, FRS:0.51, all *P* < 0.001) and MACE (ML:0.84 vs. MDI:0.82, SSS:0.76, SIS:0.73, FRS:0.53, all *P* < 0.05).

**Conclusion:** Among the patients with IS, TIA, and/or PAD, ML demonstrated a better capability of predicting ACM and MCAE than clinical scores and CCTA metrics.

## Introduction

Patients with prior history of extra-cardiac vascular diseases (EVD), such as ischemic stroke (IS), transient ischemic attack (TIA), or peripheral artery disease (PAD), have an increased risk of coronary artery disease (CAD) (1–4). The presence of EVD has been indicated to adversely affect the long-term outcome of patients, increasing the chances of myocardial infarction (MI) and non-stroke vascular death (1, 2). Once acute coronary symptom happens, patients with concurrent EVD have been reported to be associated with higher complication rates and lower procedural success rates undergoing percutaneous coronary intervention (5). Although with potentially higher CAD risk, many of them may not have a cardiac relative event (3). It is of utmost importance to identify patients with EVD who are at the highest risk of developing CAD since they require more aggressive treatment and those who received less aggressive treatment tend to have worse outcomes (6). Up to now, CAD risk stratification methods for patients with EVD have been limited since traditional cardiac scoring systems are generated from the general population (7).

Coronary computed tomography angiography (CCTA) is a non-invasive and accurate imaging modality to diagnose CAD (8), providing invaluable information for both clinical evaluation and future adverse events prediction (9). Machine learning (ML) is a promising computer science that emerges in recent years as an effective method for decision making and prediction through identifying patterns in large datasets (10). The previous study has utilized ML to predict all-cause death in patients suspected of CAD (11). However, the application of ML with CCTA data to predict cardiac relative adverse outcomes in patients with EVD has not been explored. Therefore, this study aimed to use ML with CCTA and clinical data to predict long-term cardiac relative adverse outcomes in patients with EVD.

## Methods

### Study Population

This study was approved by the institutional review board of the Guangdong Provincial People's Hospital and Guangdong Academy of Medical Sciences. Written informed consents were obtained before the CCTA scanning for all patients for this study. We evaluated consecutive patients who underwent CCTA from April 2012 to December 2018. Patients ≥18 years of age who had a documented medical history of IS, TIA, and /or PAD were eligible for inclusion. Patients who had previously diagnosed CAD (MI and/or coronary revascularization), early revascularization (within 90 days after the index CCTA), the low imaging quality of CCTA (assessed by level III radiologist), loss of follow-up, or missing data were excluded. Patients with early revascularization within 90 days after the index CCTA were excluded to avoid treatment bias (12). All eight excluded patients in the present studies underwent selective coronary intervention without acute coronary syndrome or MI.

### Clinical Data

The clinical data were obtained from the medical chart review and patient questionnaires. Hypertension was defined as systemic blood pressure >140 mmHg or currently taking anti-hypertensive medications. Smoking history was defined as current smoking or cessation of smoking within 90 days of the index CCTA examination. Diabetes mellitus (DM) was defined as the medical documentation of DM diagnosis or currently using insulin or oral hypoglycemic medications. Diabetic peripheral neuropathy was defined as a diagnosis made by a physician. A positive family history of CAD was defined as MI in first-degree family members ≤55 years of age for men and ≤65 for women. Lab tests nearest to the index CCTA examination were recorded. The Framingham risk raw score and the 10-year coronary heart disease risk [Framingham risk score (FRS)] were calculated for each patient (13). The information of statin, aspirin, and clopidogrel use before and after the index CCTA examination was obtained from the medical record or structured phone follow-up. Full details and definitions of all 35 clinical data were available in Supplementary Materials.

### Clinical Outcome

The clinical outcomes were obtained through a combination of clinical visits, medical chart reviews, and structural phone reviews by a trained research staff who was blinded to the clinical data and CCTA results. Structural phone review was performed every 6 months after the index CCTA. If patients had a regular follow-up in the clinic every 3–6 months, phone review was skipped. Medical chart review was used to obtain patient outcomes if phone review failed to contact patients, which happened in very few of our patients. The outcomes include revascularization 90 days after the index CCTA examination either by coronary artery bypass graft or percutaneous coronary intervention, unstable angina requiring hospitalization, non-fatal MI, and all-cause mortality (ACM). Major adverse cardiac events (MACE) were predefined, which was composed of unstable angina requiring hospitalization, non-fatal MI, revascularization, and ACM.

### CCTA Scanning Protocols and Imaging Reconstruction

All scans were conducted using a 128-slice dual-source scanner (SOMATOM Definition Flash; Siemens Healthineers, Forchheim, Germany). The CCTA examination was started by the injection of a bolus of 70 ml iopromide (370 mg/ml, Ulravist; Bayer, Berlin, Germany) followed by a 60 ml saline solution with an injection rate of 5 ml/s. Bolus tracking was used to control the contrast agent application with a region of interest placed in the aortic root, and the image acquisition started 5 s after the signal attenuation reached the predefined threshold of 100 Hounsfield units. A flying focal spot technique was used for the data acquisition performed with detector collimation of 2 mm × 128 mm × 0.6 mm and a gantry rotation time of 280 ms from the level of the carina to the heart base in a craniocaudal direction.

Imaging was reconstructed with the following steps. First, the best phase was automatically chosen during the 30–40% phase or 60–70% phase to obtain optimal coronary artery images. Then, for each examination, both end-systolic and end-diastolic phases were obtained to evaluate ventricular function. Imaging reconstruction was performed at 30–70% of the R-R interval in 10% increments to assess the left ventricular (LV) function. The reconstruction parameters included a section thickness of 0.6 mm with 0.5 mm increments, a Bl26 of the medium-smooth kernel, and an iterative reconstruction strength level of 5 (SAFIRE, Siemens Healthineers).

### CCTA Imaging Analysis

Coronary artery was assessed visually by level III radiologists for the degree of luminal stenosis, presence of plaque, and the composition of plaque using a 16-segment model (14). The plaque was classified as calcified, non-calcified, or mixed. The non-calcified plaque was defined as a distinct tissue structure that could be distinguished from the surrounding tissue with an area over 1 mm^2^ and density lower than the contrast-enhanced blood pool. Any plaque that has both calcified and non-calcified components was classified as mixed plaque. Luminal stenosis was classified into none; minimal (1–24%); mild (25–49%); moderate (50–69%); severe (70–99%); and occluded. Segment stenosis scores (SSS), which reflected the overall plaque extent, were calculated as the summation of the score of 16 segments. Segment involvement scores (SIS), which reflected the overall plaque distribution, were calculated by the summation of the number of segments with plaque (9). The Modified Duke index (MDI) was calculated to divide the patients into six subtypes (15). The full details of all 34 CCTA metrics are available in the Supplementary Materials.

Image datasets were imported into a dedicated workstation (Syngo.Via, Siemens Healthineers, Forchheim, Germany). The end-diastolic volume, end-systolic volume, stroke volume, ejection fraction, cardiac output, and left ventricular mass (LVM) were calculated using commercially available software (CT Cardiac Function, Syngo.Via, Siemens Healthineers, Forchheim, Germany). The contours of the endocardial and epicardial border were automated defined by the software with manual correction if necessary. The LVM was then indexed to the body surface area. An unenhanced prospectively ECG-triggered scan was used for the coronary calcium score measurement. Coronary calcifications with CT attenuation above 130 Hounsfield Units were scored using the Agatston method (16).

### Machine Learning

**Figure 1:**
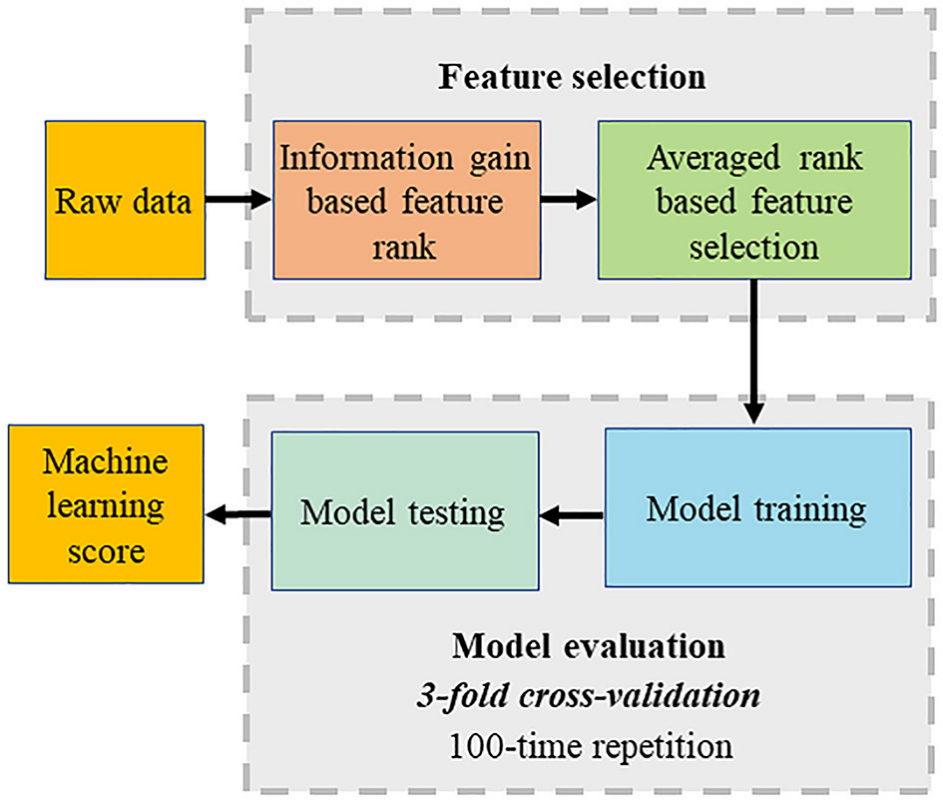
Computational methods. The machine learning (ML) process included automated feature selection by information gain ranking using four different methods, averaging the results of information gain ranking, model building (LogiBoost), 3-fold stratified cross-validation, and repletion for 100 times.

Machine learning was adopted to perform automatic feature selection, model implementation, and cross-validation. The first step was to extract relevant inputs from all the clinical and CCTA parameters as shown in Figure 1. The second step was to use a boosted ensemble algorithm to build the prediction model. While the last step was to adopt 3-fold cross-validation for a statistically significant evaluation. All steps were implemented using the open-source Waikato Environment for Knowledge Analysis platform (17).

The feature selection was implemented using “information gain attribute ranking” (18) to evaluate the relevance of an attribute with the prediction of the training data. Information gain is a metric to evaluate the relevance of an attribute with the prediction of the training data. Attributes with an information gain no larger than 0 are not relevant to the prediction, which will increase the dimension of the model and even degrade the prediction accuracy. Thus, only attributes with an information gain larger than 0 were selected to build a prediction model. Four different methods of information gain were performed, which included histogram binning (19), Platt scaling (20), isotonic regression (21), and Bayesian Binning into Quantiles (22). The average information gain ranking calculated from four methods was used for ML model building.

The model implementation employed an ensemble classification approach, LogitBoost, to make reliable predictions, which combines a set of weak base predictors to make a single strong predictor by iteratively updating their appropriate weighting according to their performance. The intuition is that each weak base predictor has its unique strengths and weaknesses (e.g., it performs poorly in some data), and their combination using specific weighting can foster strengths and circumvent weaknesses to achieve high performance. When the training is completed, a set of base predictions is the weighted results of the base prediction models using the weighting distribution.

Cross-validation was used to evaluate the process including feature selection and model implementation considering the limited data for training and testing. Compared with the conventional split strategy, the main advantage is the reduced variance in the prediction error resulting in a more reliable evaluation and a maximized use of all the data to make an accurate overall evaluation. Particularly, 3-fold cross-validation was adopted. The dataset was divided into three equal folds, each with almost the same number of events. Then, three validation experiments were performed, and in each experiment, each fold was used as the validation dataset and others as the training dataset. Thus, each data was used for validation once. Finally, the above process was repeated 100 times, and the final validation performance was the average of all the validation results.

### Statistics Analysis

Continuous variables are presented as mean ± SD and categorical variables are presented as exact numbers (frequency). Receiver-operating characteristic (ROC) analysis and pairwise comparisons were used to compare ML with the clinical risk score (FRS) and CCTA data (MDI, SSS, and SIS) for the diagnostic performance (23).

## Result

### Study Population

**Figure 2:**
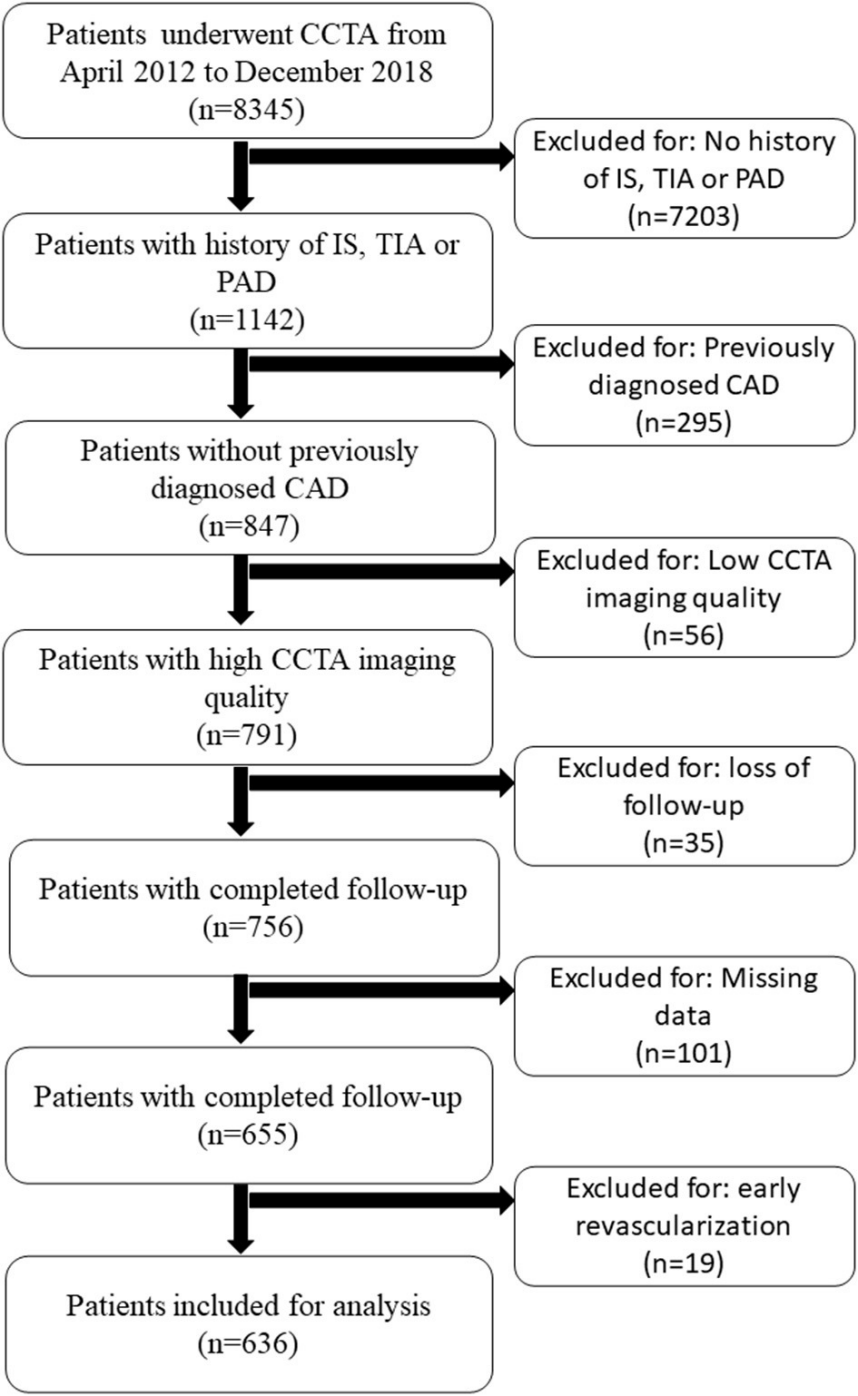
Patient selection flow chart. CAD, coronary artery disease; CCTA, coronary computed tomography; IS, ischemic stroke; PAD, peripheral artery disease; TIA, transient ischemic attack.

Of 8,345 consecutive patients who underwent CCTA for suspected CAD, 1,142 had a history of IS, TIA, and/or PAD. A total of 636 patients with EVD with full clinical and CCTA data were included. The detailed patient inclusion and exclusion flow chart is demonstrated in Figure 2. The average age was 67 ± 11 years (range from 32 to 89). Sixty-four percent of the patients were men (406 of 636). Overall, 408 (64.2%) patients had a history of IS, 71 (11.2%) patients had a history of TIA, and 77 (12.1%) patients had a history of PAD. The rest of the patients had at least two of the above EVD history. A minority of patients took cardiovascular protective medications before the index CCTA examination. The percentage increased dramatically after the index CCTA examination. On the CCTA analysis, 165 (25.9%) patients had no stenosis, 125 (19.7%) had minimal stenosis, and 135 (21.2%) had mild stenosis. Of the remaining 211 patients with obstructive disease (stenosis over 50%), 113 (17.8%) had moderate stenosis, 97 (15.3%) had severe stenosis, and one had an occluded segment (first diagonal branch). All clinical characteristics are described in Table 1.

**Table 1:** Clinical and coronary CT angiography (CCTA) characteristics.

**Characteristic**	**Data** **(*n* = 636)**
Age (years ± SD)	67 ± 11
**Sex**, ***n*** **(%)**	
Male	406 (63.8)
Female	230 (36.2)
BMI	24.03 ± 3.1
**Patient composition**, ***n*** **(%)**	
IS	408 (64.2)
TIA	71 (11.2)
PAD	77 (12.1)
IS and TIA	15 (2.3)
IS and PAD	58 (9.1)
TIA and PAD	3 (0.5)
IS, TIA, and PAD	4 (0.6)
**CAD risk factors**, ***n*** **(%)**	
Hypertension	434 (68.2)
Diabetes	228 (35.8)
Dislipidemia	263 (41.4)
Hyperuricemia	326 (51.3)
Familial history of CAD	61 (9.6)
Current smoking	109 (17.1)
Symptoms, *n* (%)
No chest pain	310 (48.7)
Chest pain	326 (51.2)
Chest pain on exertion	115 (18.1)
Chest pain relief with GTN	51 (8.0)
Dyspnea on exertion	103 (16.2)
**Medication before index CCTA examination**, ***n*** **(%)**	
Aspirin	38 (5.9)
Statin	40 (6.2)
P2Y12 inhibitors	25 (3.9)
**Medication after index CCTA examination**, ***n*** **(%)**	
Aspirin	232 (36.5)
Statin	429 (67.4)
P2Y12 inhibitors	198 (31.1)
**Coronary artery calcium score**, ***n*** **(%)**	
0	201 (31.6)
0.1–100	194 (30.5)
101–400	134 (21.1)
>400	107 (16.8)
**Obstructive CAD severity**, ***n*** **(%)**	
No stenosis	165 (25.9)
Minimal stenosis	125 (19.7)
Mild stenosis	135 (21.2)
Moderate stenosis	113 (17.8)
Severe stenosis	97 (15.3)
Totally occluded	1 (0.1)
**Duke prognostic CAD risk index**, ***n*** **(%)**	
1	403 (63.4)
2	110 (17.3)
3	46 (7.2)
4	55 (8.6)
5	2 (0.3)
6	20 (3.1)
**Number of involved vessels** **≥50% stenosis**, ***n*** **(%)**	
Left main CAD	21 (3.3)
Single-vessel CAD	118 (18.6)
Two-vessel CAD	52 (8.2)
Three-vessel CAD	20 (3.1)

*BMI, body mass index; CAD, coronary artery disease; CCTA, coronary computed tomography angiography; GTN, glyceryl trinitrate; IS, ischemic stroke; PAD, peripheral artery disease; TIA, transient ischemic attack*.

The mean follow-up time was 3.9 ± 1.6 years. During the follow-up, 21 patients had at least one episode of unstable angina requiring hospitalization, eight patients had non-fatal MI, 23 patients had revascularization 90 days after the index CCTA, and 13 patients died.

### Feature Selection

**Figure 3:**
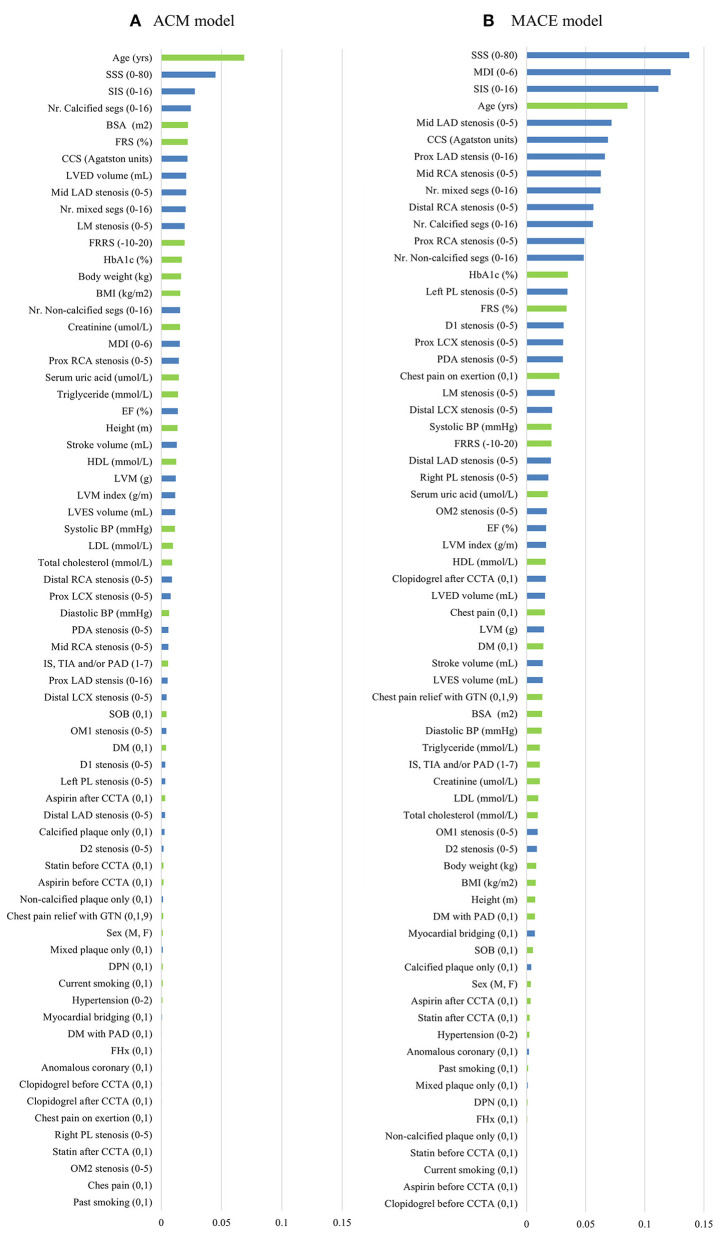
Feature selection of two models. Thirty-five CT angiography metrics (blue) and 34 clinical variables (green) were available. The information gain ranking was to evaluate the relevance of an attribute with the prediction of the training data. Four different methods of information gain ranking were used for each model. This figure shows the average results of four different methods for ACM model **(A)** and MACE model **(B)**, respectively. ACM, all-cause mortality; BMI, body mass index; BP, blood pressure; BSA, body surface area; CCS, coronary calcium score; CCTA, coronary computed tomography angiography; D, diagonal; DM, diabetes mellitus; DPN, diabetic peripheral neuropathy; EF, ejection fraction; FHx, family history; FRRS, Framingham risk raw score; FRS, Framingham risk score; GTN, glyceryl trinitrate; HbA1c, hemoglobulin A1c; HDL, high-density lipoprotein; IS, ischemic stroke; LAD, left anterior descending artery; LCX, left circumflex; LDL, low-density lipoprotein; LM, left main; LVED, left ventricular end diastolic; LVES, left ventricular end systolic; LVM, left ventricular mass; MACE, major adverse cardiac events; MDI, modified Duke index; Nr., number; Mid, middle; OM, obtuse marginal; PAD, peripheral artery; PL, posterolateral branch; prox, proximal; RCA, right coronary artery; segs, segments; SIS, segment involvement score; SOB, shortness of breath; SSS, segment stenosis score; TIA, transient ischemic stroke.

Figure 3 shows the result of the information gain, ranking from high to low. For the ACM prediction, age was the number one top-ranked feature. Importantly, in the top 10 ranked features, seven were CCTA data. For the MACE prediction, the highest-ranked feature was SSS. In addition, nine of the 10 top-ranked features were CCTA data.

### Prediction of Long-Term Adverse Events

**Figure 4:**
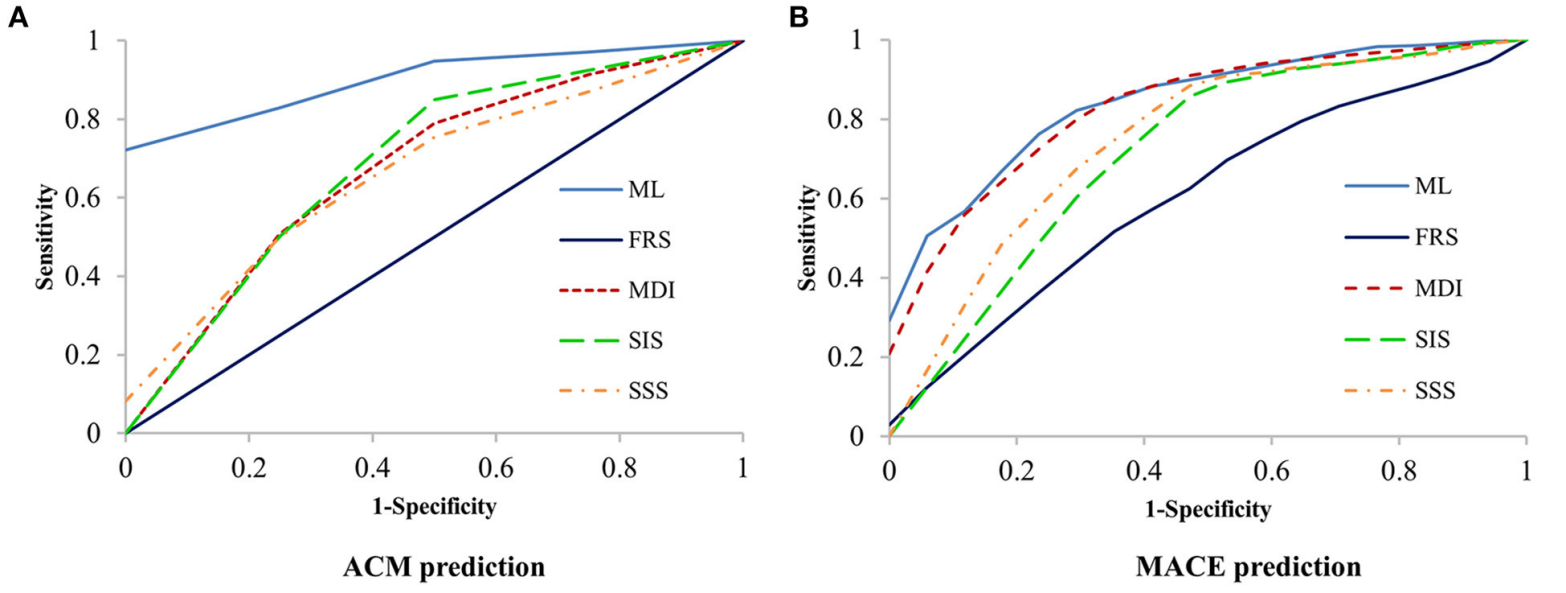
Receiver-operating characteristic (ROC) curves for prediction of ACM and MACE. Machine learning had a significantly higher area-under-the curve for prediction of ACM (*P* < 0.001, **A**) and MACE (*P* < 0.05, **B**) than all other scores. ACM, all-cause mortality; FRS, Framingham risk score; MACE, major adverse cardiac events; MDI, modified Duke index; SIS, segment involvement score; SSS, segment stenosis score.

Machine learning demonstrated a significant higher area under the curve (AUC) compared with clinical risk score and CCTA data alone for the prediction of ACM (ML: 0.92 vs. MDI: 0.66, SSS: 0.68, SIS: 0.67, FRS: 0.51, *P* < 0.001 for all) (Figure 4A) and (ML: 0.84 vs. MDI: 0.82, SSS: 0.76, SIS: 0.73, FRS: 0.53, *P* < 0.05 for all) (Figure 4B). Tables 2, 3 show the detailed accuracy, sensitivity, specificity, positive predictive value (PPV), and negative predictive value (NPV) for ML, FRS, MDI, SSS, and SIS for the prediction of ACM and MACE, respectively. As shown in Table 2, we can notice that the accuracy, sensitivity, specificity, and PPV of our ML method are much higher than others for the prediction of ACM, and the NPV of our ML method is slightly higher than that of others. We can also notice that the accuracy and specificity are almost the same, which is due to the fact that normal (negative) cases make up the majority of the dataset. The same pattern can be also observed in Table 3 but with a much smaller improvement for the diagnostic performance of our ML method compared with others. Even though the patient population we studied is at a high risk of CAD with an event rate of around 10% higher than the general population, this is still statistically low, which could explain the low PPV (24).

**Table 2:** Accuracy, sensitivity, specificity, positive predictive value, and negative predictive value of our ML method and existing methods for prediction of all-cause mortality.

	**Accuracy**	**Sensitivity**	**Specificity**	**PPV**	**NPV**
ML	0.811	0.846	0.811	0.085	0.996
FRS	0.511	0.538	0.510	0.022	0.981
MDI	0.610	0.615	0.610	0.032	0.987
SSS	0.631	0.615	0.631	0.034	0.987
SIS	0.619	0.615	0.620	0.033	0.987

*FRS, Framingham risk score; MDI, modified Duke index; ML, machine learning; NPV, negative predictive value; PPV, positive predictive value; SIS, segment involvement score; SSS, segment stenosis score*.

**Table 3:** Accuracy, Sensitivity, specificity, positive predictive value, and negative predictive value of our ML method and existing methods for prediction of major adverse cardiac events.

	**Accuracy**	**Sensitivity**	**Specificity**	**PPV**	**NPV**
ML	0.750	0.745	0.750	0.220	0.969
FRS	0.520	0.527	0.520	0.094	0.921
MDI	0.741	0.745	0.740	0.214	0.968
SSS	0.701	0.709	0.701	0.183	0.962
SIS	0.679	0.673	0.680	0.166	0.956

*FRS, Framingham risk score; MDI, modified Duke index; ML, machine learning; NPV, negative predictive value; PPV, positive predictive value; SIS, segment involvement score; SSS, segment stenosis score*.

## Discussion

In the current study, we explored the ability of ML to predict the long-term prognosis of patients with EVD using both clinical and CCTA data. We found ML to be effective in the prediction of ACM and MACE compared with the traditional risk score and CCTA data alone. This suggests the great potential of ML in the prediction of long-term prognosis in patients suspected with CAD who had a history of EVD.

The risk of CAD in patients with IS, TIA, and/or PAD is high since they are epidemiologically and pathophysiologically commonly relative (2–4, 6, 25). However, a considerable number of them may not even have a cardiac relative event (3). More importantly, patients with EVD often received more intensive medical treatment at baseline. But when an acute coronary event occurs, they tend to end up with worse outcomes (6), which suggests that the current treatment strategy might be insufficient for this type of patient. It is, therefore, of critical importance to identify patients with the highest risk that may benefit from more intensified prevention strategies (26). In addition, once precise risk stratification is available, a new treatment strategy could be applied to explore the most optimal treatment plan for these high-risk patients. However, individual risk assessment using comprehensive risk scores (13, 27) is not satisfactory as the mechanisms of cardiovascular events are far more intricate (28). Importantly, the medication use of patients after a first vascular event may change the influence of those risk factors. This has been supported by the Framingham cohort that patients with CAD or stroke had a lower number of risk factors associated with coronary events (29). Coronary CTA has great value for CAD risk stratification (30, 31) but traditional methods usually analyze a single metric at a time, for instance, luminal stenosis severity, plaque burden, or coronary calcium content alone, leaving other important variables unexplored (32). ML is a promising method that can overcome these limitations by integrating numerous risk factors.

Machine learning demonstrated the highest performance for risk prediction in patients with EVD, which showed the highest accuracy, sensitivity, specificity, PPV, and AUC compared with other conventional methods. Both for the ACM and MACE prediction, FRS showed the lowest diagnostic value (sensitivity, specificity, PPV, accuracy, and AUC). One reason is that FRS is developed for the general population risk stratification and may be inferior for coronary artery-related event prediction in patients with EVD. Another possible reason is that FRS only includes a limited number of previously well-known risk factors of CAD, with many others that may contribute significantly not taken into consideration. MDI, SSS, and SIS are all CCTA metrics that reveal the stenosis of the coronary artery tree. They showed a moderately high diagnostic value for event prediction. However, they do not take into consideration other important clinical data (e.g., age, sex, BMI, medical symptoms) and cardiac metrics (e.g., LVM and cardiac output). It is difficult for clinicians to take an overall consideration of numerous CCTA variables and clinical data for patient risk-stratification, which has been overcome by ML. Furthermore, we found that many risk factors that have not been taken into consideration in FRS or CCTA risk assessing systems, such as HbA1c, serum uric acid, and LVM (Figure 3), showed significant contributions in the ML model. By providing deep integration of CCTA data and clinical risk factors, ML provided the highest prediction capability compared with others.

We used information gain attribute ranking for risk assessment. This approach avoided the preconception of previous medical knowledge which could identify unexpected but important risk factors. In the present study, body surface area (BSA) was the top five ranked feature in the ACM model. A previous study had indicated that BSA was associated with ACM and cardiovascular-related death in patients with heart failure (33). Another study had developed a novel metric called body shape index, calculated using BSA, to successfully predict ACM in a large population of 11,808 (34). Our result suggested that BSA may be an important prognostic factor for ACM prediction in patients with EVD. In addition, the importance of CCTA has been addressed since CCTA metrics ranked very high, especially for the MACE model.

The present study had the following strengths. First, we explored more cardiac-relative endpoints. Second, we included the use of cardiovascular protective medications in building the ML model for prediction. A considerable number of patients who did not take cardiovascular protective medications started taking single or multiple medications after the index CCTA examination. These medications may have a certain effect on the long-term prognosis. Third, we included more clinical data and CCTA metrics such as HbA1c, diabetes peripheral neuropathy, serum uric acid, BSA, LVM, and LVM index in the building of the ML model. All of these had not been used in the study of Motwani (11).

### Study Limitations

This is a single-center observational study with a small number of patients. A large-scale study is required to provide stronger evidence. Nevertheless, the result of the current study was in alignment with the finding of the study of Motwani in terms of the prediction of ACM (11), even though we focused on a different patient population. Second, the conventional systems that we used for comparison were developed mainly on a naive population for event prediction and therefore may underestimate the CAD risk for patients with EVD. However, they are the commonly used clinical or imaging risk stratification systems and are considered the best representatives under the condition that no other existing system is developed for this patient population. In addition, all patients were recommended for CCTA examination by clinical physicians, which represented real-world clinical practice. Another limitation is that even though during follow-up, we would like to differentiate the cause of death into cardiac death and non-cardiac death. However, it was difficult to ascertain the cause of death for some patients without medical documentation. To avoid the misclassification of the cause of death in some patients, we used ACM. Plus, we excluded patients with early elective revascularization after CCTA to avoid treatment bias. This, in another way, made the result of our study artificial and far from current cardiology decision-making. Finally, for patients with fewer clinical or CCTA data than those included in the present study, the ML model we built should not be used since it may not be accurate. However, all the CCTA metrics could be obtained with a single standard CCTA scan. In addition, it is reasonable to have a thorough lab assessment for patients with EVD suspected with CAD.

## Conclusions

The result of the present study demonstrated that it was feasible and efficient to predict ACM and MACE in patients with a previous history of ECV using the ML model with clinical and CCTA metrics.

## Data Availability Statement

The datasets presented in this study can be found in online repositories. The names of the repository/repositories and accession number(s) can be found below: https://github.com/XiaoweiXu/LogitBoost_Prediction.

## Ethics Statement

The studies involving human participants were reviewed and approved by the Institutional Review Board of Guangdong Provincial People's Hospital and Guangdong Academy of Medical Sciences. The patients/participants provided their written informed consent to participate in this study.

## Author Contributions

GL, XZ, YX, TL, YY, MC, CL, and YL contributed to data collection. QL, YC, AZ, and XX contributed to data analysis. GL, XZ, and XX contributed to writing the manuscript. MH and XX contributed to writing and critically revising the manuscript. All authors participated in the study design and approved the final draft.

## Funding

This work was supported by the Science and Technology Planning Project of Guangdong Province under Grant Nos. 2017A070701013, 2017B090904034, 2017B030314109, 2018B090944002, 2019B020230003, and 2019A050510041, Guangdong peak project under Grant No. DFJH201802, the National Key Research and Development Program under Grant No. 2018YFC1002600, the Natural Science Foundation of Guangdong Province under Grant No. 2018A030313785, the Natural Science Foundation of China under Grant No. 62006050, and Sanming Project of Medicine in Shenzhen (No. SZSM202011005).

## Conflict of Interest

The authors declare that the research was conducted in the absence of any commercial or financial relationships that could be construed as a potential conflict of interest.

## Publisher's Note

All claims expressed in this article are solely those of the authors and do not necessarily represent those of their affiliated organizations, or those of the publisher, the editors and the reviewers. Any product that may be evaluated in this article, or claim that may be made by its manufacturer, is not guaranteed or endorsed by the publisher.

## Abbreviations

ACM: all-cause mortality
BSA: body surface area
CAD: coronary artery disease
CCTA: coronary computed tomography angiography
EVD: extra-cardiac vascular disease
FRS: Framingham risk score
IS: ischemic stroke
LVM: left ventricular mass
MACE: major adverse cardiac events
MDI: modified Duke index
MI: myocardial infarction
ML: machine learning
NPV: negative predictive value
PAD: peripheral artery
PPV: positive predictive value
SIS: segment involvement score
SSS: segment stenosis score
TIA: transient ischemic stroke.
