# Supplementary material for: Machine Learning to Predict Long-Term Cardiac-Relative Prognosis in Patients With Extra-Cardiac Vascular Disease
Source: Front Cardiovasc Med. 2021 Nov 25;8:771504. doi: 10.3389/fcvm.2021.771504 (PMC8655836; doi:10.3389/fcvm.2021.771504)
Supplement: Supplementary file 1 [file Data_Sheet_1.DOCX]

**Appendix**

Explanatory notes for all clinical and CCTA data in the present study (as listed in Figure 3), are given below:

**Clinical Explanatory Notes**

Age (yrs) Age at time of CCTA.

Sex (M, F) M = Male, F = Female.

IS, TIA or PAD (1-7) Ischemic stroke (IS), transient ischemic stroke (TIA), or peripheral artery disease (PAD). 1 = IS only, 2 = TIA only, 3 = PAD, 4 = IS and TIA, 5 = IS and PAD, 6 = TIA and PAD, 7 = IS, TIA, and PAD

Current smoking (0,1) Smoking within 3 months. 0 = No, 1 = Yes.

Past smoking (0,1) Cessation >3 months. 0 = No, 1 = Yes.

DM (0,1) Diabetes Mellitus. Hemoglobulin A1c≥6.5% or fasting plasma glucose≥7.0mmol/L or 2 hour plasma glucose ≥11.1mmlol/L during oral glucose tolerance test. 0 = No, 1 = Yes.

DM with PAD (0,1) Diabetes Mellitus with peripheral artery disease. 0 = No, 1 = Yes. Peripheral artery disease is considered present if the ankle-brachial index was 0.90 or less, if it is documented in the medical system or if there is a history of limb revascularization.

DPN (0,1)  Diabetic peripheral neuropathy. Abnormal never conduction test or 10 g monofilament test or a documented history in the medical system. 0 = No, 1 = Yes.

HbA1c (%) Hemoglobulin A1c as %. Blood test nearest to the index examination.

Hypertension (0,1) Documented history of systolic blood pressure >140mmHg or treatment with anti-hypertensive medications. 0 = No, 1 = Yes.

Systolic BP (mmHg) Systolic blood pressure nearest to the index examination.

Diastolic BP (mmHg) Diastolic blood pressure nearest to the index examination.

FHx (0,1) Family history of premature coronary heart disease was defined as history of myocardial infarction of a first-degree relative below the age of 55 years for male and 65 years for female relatives. 0 = No, 1 = Yes.

SOB (0,1) Shortness of breath. Patient self-report as documented in the medical system. 0 = No, 1 = Yes.

Chest pain (0,1) Patient self-report as documented in the medical system. 0 = No, 1 = Yes.

Chest pain with exertion (0,1) Patient self-report as documented in the medical system. 0 = No, 1 = Yes.

Chest pain relief with GTN (0,1) Patient self-report as documented in the medical system. 0 = No, 1 = Yes.

Creatinine (umol/L) Blood test nearest to the index examination.

Serum uric acid (umol/L) Blood test nearest to the index examination.

Body weight (kg) Measurement nearest to the index examination.

Height (m) Measurement nearest to the index examination.

BMI (kg/m2) Body Mass Index.

BSA (m2) Body Surface Area.

Total cholesterol (mmol/L) Blood test nearest to the index examination.

HDL (mmol/L) High-density lipoprotein. Blood test nearest to the index examination.

LDL (mmol/L) Low-density lipoprotein. Blood test nearest to the index examination.

Triglyceride (mmol/L) Blood test nearest to the index examination.

FRS (%) Framingham Risk Score as %.

FRRS (-10-20) Framingham risk raw score.

Aspirin before CCTA (0,1) Regularly taking aspirin before the index examination.

0 = No, 1 = Yes.

Aspirin after CCTA (0,1) Regularly taking aspirin after the index examination.

0 = No, 1 = Yes.

Statin before CCTA (0,1) Regularly taking statin before the index examination.

0 = No, 1 = Yes.

Statin after CCTA (0,1) Regularly taking statin after the index examination.

0 = No, 1 = Yes.

Clopidogrel before CCTA (0,1) Regularly taking clopidogrel before the index examination.

0 = No, 1 = Yes.

Clopidogrel after CCTA (0,1) Regularly taking clopidogrel after the index examination.

0 = No, 1 = Yes.

**CCTA**

CCS (Agatston units) Coronary Calcium Score.

LM stenosis (0-5) Left main

Prox LAD stenosis (0-5) Proximal left anterior descending

Mid LAD stenosis (0-5) Mid left anterior descending

Distal LAD stenosis (0-5) Distal left anterior descending

D1 stenosis (0-5) First diagonal branch

D2 stenosis (0-5) Second diagonal branch

Luminal diameter stenosis visually scored as:

0 (none, 0%),

1 (minimal, 1-24%),

2 (mild, 25-49%),

3 (moderate, 50-69%),

4 (severe, 70-99%),

5 (total occluded).

Prox LCX stenosis (0-5) Proximal left circumflex

Distal LCX stenosis (0-5) Distal left circumflex

OM1 stenosis (0-5) First obtuse marginal branch

OM2 stenosis (0-5) Second obtuse marginal branch

Left PL stenosis (0-5) Left posterolateral branch

Right PL stenosis (0-5) Right posterolateral branch

PDA stenosis (0-5) Posterior descending artery

Prox RCA stenosis (0-5) Proximal right coronary artery

Mid RCA stenosis (0-5) Mid right coronary artery

Distal RCA stenosis (0-5) Distal right coronary artery

Anomalous coronary (0,1) 0 = No, 1 = Yes.

Myocardial bridging (0,1) 0 = No, 1 = Yes.

Mixed plaques only (0,1) 0 = No, 1 = Yes.

Calcified plaque only (0,1) 0 = No, 1 = Yes.

Non-calcified plaque only (0,1) 0 = No, 1 = Yes.

Nr. calcified segs (0-16) Number of coronary segments with calcified plaque.

Nr. non-calcified segs (0-16) Number of coronary segments with non-calcified plaque.

Nr. mixed segs (0-16) Number of coronary segments with mixed plaque.

EF % on CT (%) Left ventricular ejection fraction by CT.

LVED volume (mL) Left ventricular end-diastolic volume by CT.

LVES volume (mL) Left ventricular end-sistolic volume by CT.

Stroke volume (mL) Stroke volume by CT.

LVM (g) Left ventricular mass by CT.

LVMI (g/m) Left ventricular mass index.

SSS (0-80) Segment Stenosis Score. For each patient, individual coronary segments (16-segment model) were scored 0 to 5 based on luminal diameter stenosis, and then summed to yield a total SSS (0 to 80).

SIS (0-16) Segment Involvement Score. Summation of the absolute number of coronary artery segments with plaque, irrespective of the degree of luminal stenosis (0 to 16)

MDI (1-6) Modified Duke prognostic CAD index (MDI). Categorizes patients into the following subsets, each with an increasing risk of 5-year death: 1) <50% stenosis; 2) ≥2 stenoses 30-49% (including 1 artery with proximal disease) or 1 vessel with 50-69% stenosis; 3) 2 stenoses 50-69% or 1 vessel with ≥70% stenosis; 4) 3 stenoses 50-69% or 2 vessels with ≥70% stenosis or proximal left anterior descending stenosis ≥70%; 5) 3 vessels ≥70% stenoses or 2 vessels ≥70% stenosis with proximal LAD; and 6) LM stenosis ≥50%
